# Supplementary material for: Genetic architecture of the murine serum metabolome reveals carboxyl esterases as master regulators of circulating fatty acid metabolism
Source: PLoS Genet. 2026 Nov 2;22(11):e1012223. doi: 10.1371/journal.pgen.1012223 (PMC13750656; doi:10.1371/journal.pgen.1012223)
Supplement: Supplementary file 1 [file pgen.1012223.s001.pdf]

S1 Appendix for

Genetic architecture of the murine serum metabolome reveals the carboxyl esterases as master regulators of circulating fatty acid metabolism

Gregory R. Keele,[1,2] Travis Nemkov,[3,4] Ariel M. Hay,[5] Matthew Vincent,[2] Callan O'Connor,[2] Daniel Stephenson,[3] Grier P. Page,[1] James C Zimring,[6,7] Gary A. Churchill,[2,*] Angelo D'Alessandro[3,4,*]

1) RTI International, Research Triangle Park, NC, USA;
2) The Jackson Laboratory, Bar Harbor, ME, 04609, USA;
3) Department of Biochemistry and Molecular Genetics, University of Colorado Denver – Anschutz Medical
4) Campus, Aurora, CO, USA;
5) Omix Technologies Inc, Aurora, CO, USA;
6) Department of Pathology, University of Virginia, Charlottesville, VA, USA;
7) Department of Pathology and Laboratory Medicine, University of British Columbia, Vancouver, BC, Canada;
8) Canadian Blood Services, Ottawa, ON, Canada

***Corresponding authors:**
Gary A. Churchill, PhD
The Jackson Laboratory
Bar Harbor, ME, USA.
E-mail: gary.churchill@jax.org

Angelo D'Alessandro, PhD ***lead contact**
Department of Biochemistry and Molecular Genetics
University of Colorado Anschutz Medical Campus
12801 East 17th Ave., Aurora, CO 80045
Phone # 303-724-0096
E-mail: angelo.dalessandro@cuanschutz.edu
www.dalessandrolab.com

TABLE OF CONTENTS

SUPPLEMENTARY FIGURES 2
FIGURE A in S1 Appendix........ 2
FIGURE B in S1 Appendix........ 3
FIGURE C in S1 Appendix........ 4
FIGURE D in S1 Appendix........ 5
FIGURE E in S1 Appendix........ 6
FIGURE F in S1 Appendix........ 7
FIGURE G in S1 Appendix........ 8

SUPPLEMENTARY DATA TABLES .... XLSX FILES
SUPPLEMENTARY DATA S1 ....S1
SUPPLEMENTARY DATA S2 ....S2
SUPPLEMENTARY DATA S3 ....S3
SUPPLEMENTARY DATA S4 ....S4

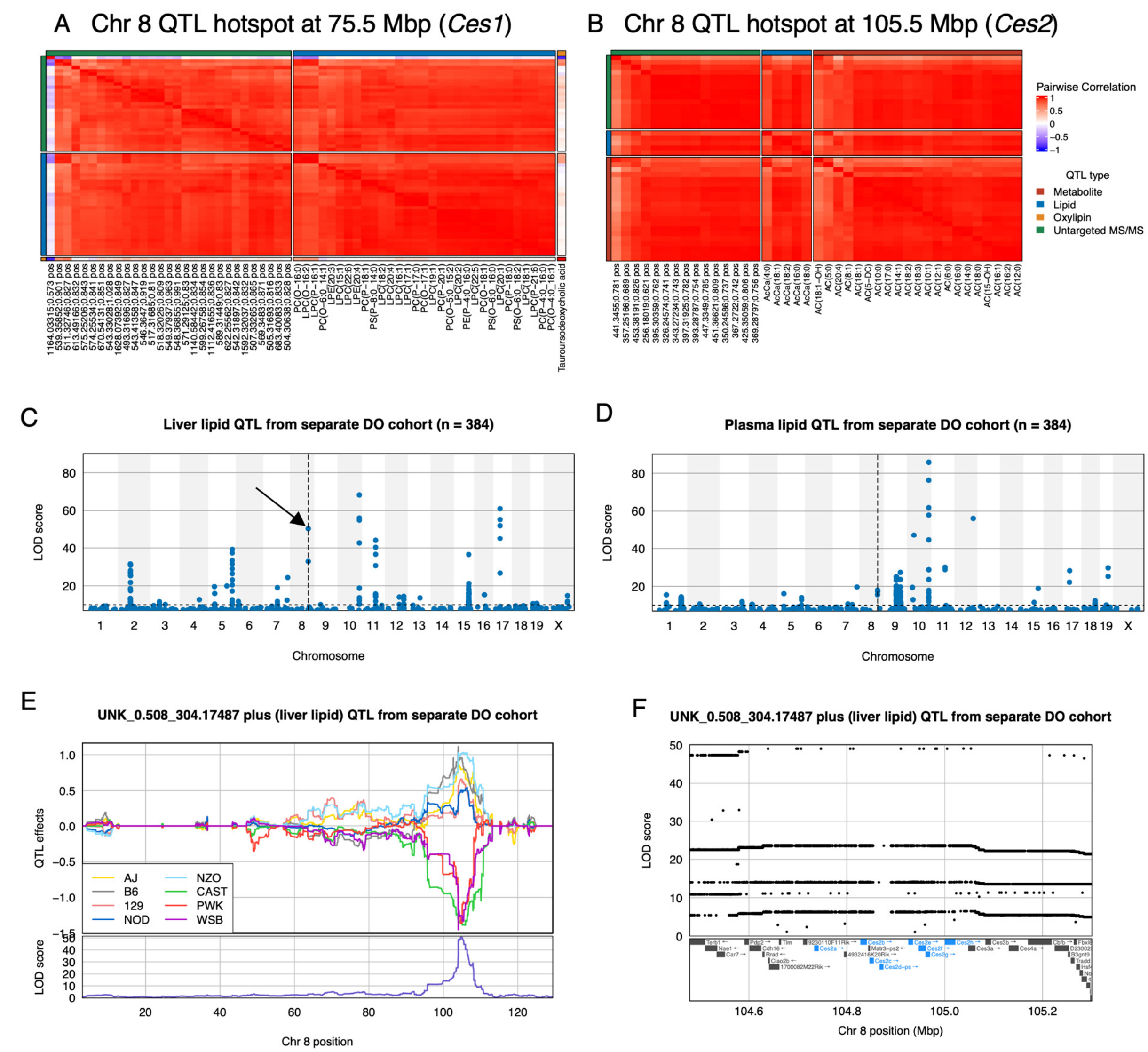

Figure A. Replication of lipid and carnitine QTL at Ces2 in a separate DO cohort, related to Figure 3. The heatmaps of the correlation matrix of haplotype effects for the QTL mapping to (A) the hotspot at 75.5 Mbp and (B) the hotspot at 105.5 Mbp. Manhattan plots of detected QTL (LOD score > 7) for (C) liver lipids and (D) plasma lipids in a separate DO cohort of 384 mice. Vertical dashed lines represent the *Ces2* locus. Horizontal dashed line represents a LOD score threshold of 10, used to define QTL hotspots in study cohort. Black arrow highlights QTL used in Figure S1E,F. (E) Aligned haplotype effects and genome scan on chromosome 8 for unidentified liver lipid (UNK_0.508_304.17487 plus). (F) SNP association and genes within the QTL 1.5 LOD drop support interval for unidentified liver lipid (UNK_0.508_304.17487 plus). Candidate genes of interest highlighted in blue. Annotations for gene models were removed for clarity. Genomic coordinates for QTL, genes, and SNPs are from GRCm38.

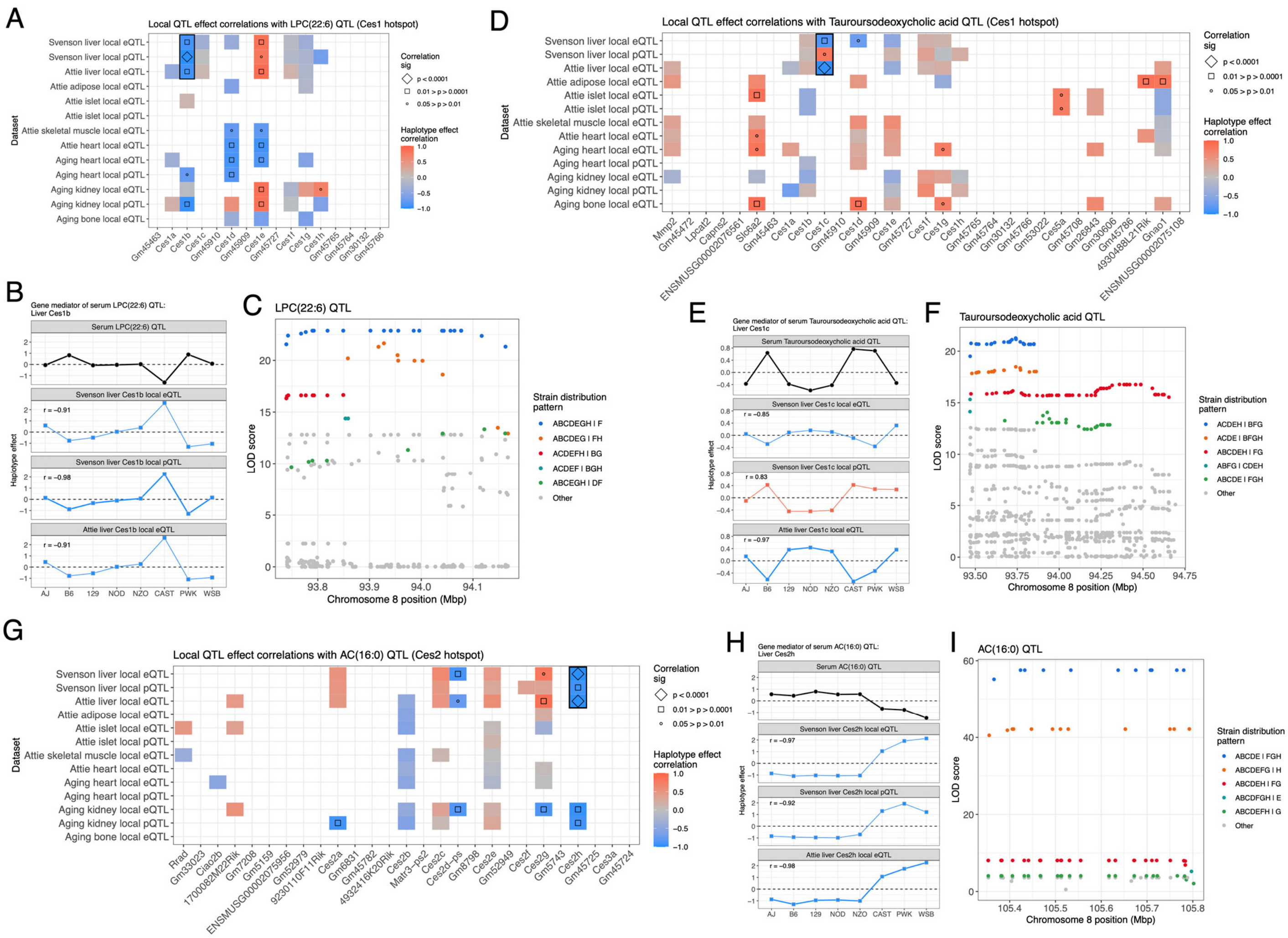

Figure B. Integration of local eQTL and pQTL to prioritize gene candidates at *Ces1* and *Ces2* hotspots, related to Figure 3. (A) Correlation of haplotype effects at local eQTL and pQTL from publicly available DO datasets with the LPC(22:6) QTL (Ces1 hotspot). Black box indicates local eQTL and pQTL highlighted in (B). Line connecting haplotype effect point estimates included to emphasize consistent or inverted effect patterns. (C) SNP association within the LPC (22:6) QTL 1.5 LOD support interval with the top five strain distribution patterns highlighted. (D) Correlation of haplotype effects at local eQTL and pQTL from publicly available DO datasets with the tauroursodeoxycholic acid QTL (Ces1 hotspot). Black box indicates local eQTL and pQTL highlighted in (E). Line connecting haplotype effect point estimates included to emphasize consistent or inverted effect patterns. (F) SNP association within the tauroursodeoxycholic acid QTL 1.5 LOD support interval with the top five strain distribution patterns highlighted. (G) Correlation of haplotype effects at local eQTL and pQTL from publicly available DO datasets with the AC(16:0) (Ces2 hotspot). Black box indicates local eQTL and pQTL highlighted in (H). Line connecting haplotype effect point estimates included to emphasize consistent or inverted effect patterns. (I) SNP association within the tauroursodeoxycholic acid QTL 1.5 LOD support interval with the top five strain distribution patterns highlighted. Strain distribution pattern coding: AJ – A, B6 – B, 129 – C, NOD – D, NZO – E, CAST – F, PWK – G, WSB – H.

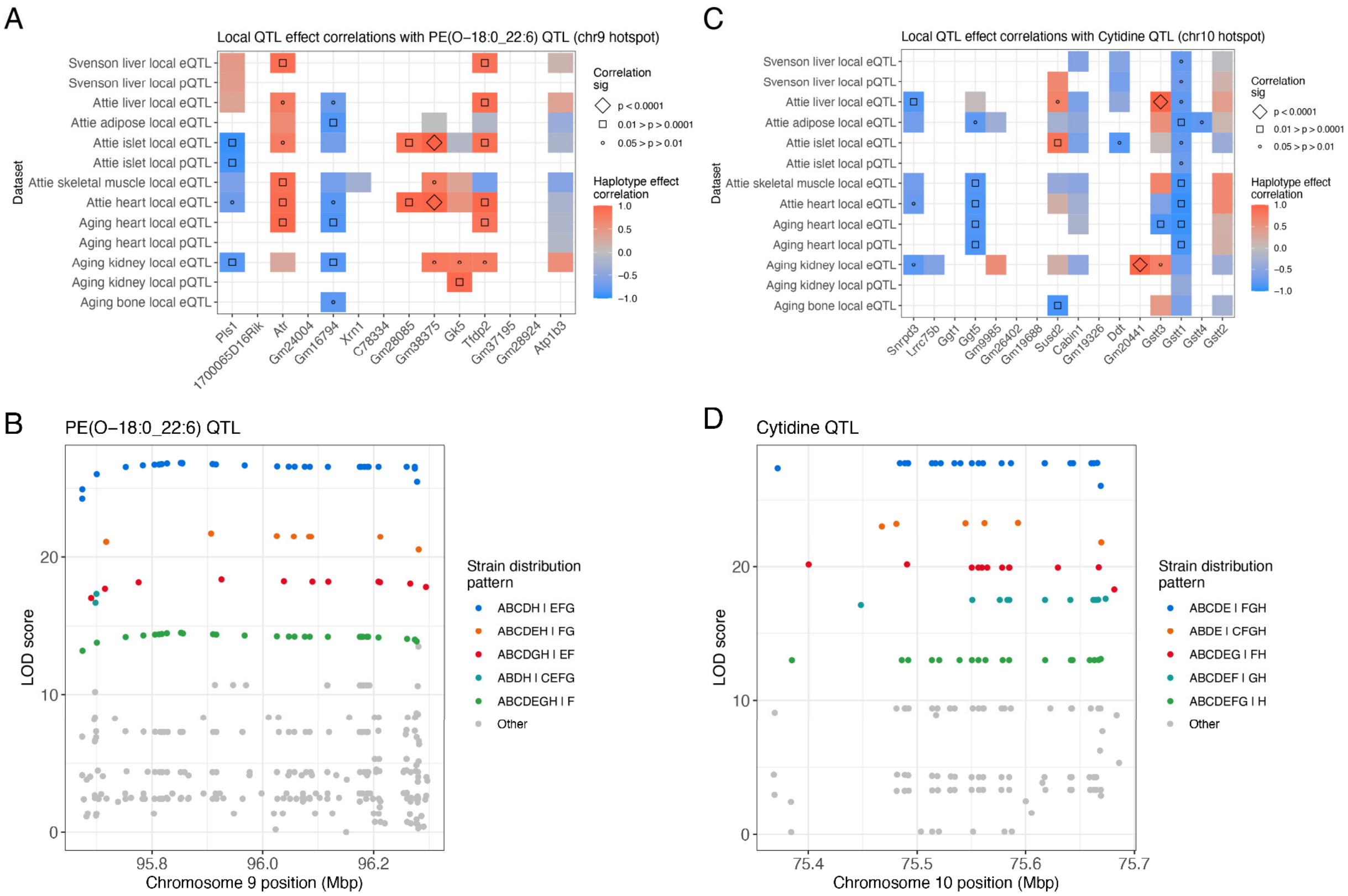

Figure C. Integration of local eQTL and pQTL to prioritize gene candidates at chromosomes 9 and 10 hotspots, related to Figures 4 and 5. (A) Correlation of haplotype effects at local eQTL and pQTL from publicly available DO datasets with the PE(O—18:0_22:6) QTL (chromosome 9). (B) SNP association within the PE(O—18:0_22:6) QTL 1.5 LOD support interval with the top five strain distribution patterns highlighted. (C) Correlation of haplotype effects at local eQTL and pQTL from publicly available DO datasets with the cytidine QTL (chromosome 10). (D) SNP association within the cytidine QTL 1.5 LOD support interval with the top five strain distribution patterns highlighted. Strain distribution pattern coding: AJ – A, B6 – B, 129 – C, NOD – D, NZO – E, CAST – F, PWK – G, WSB – H.

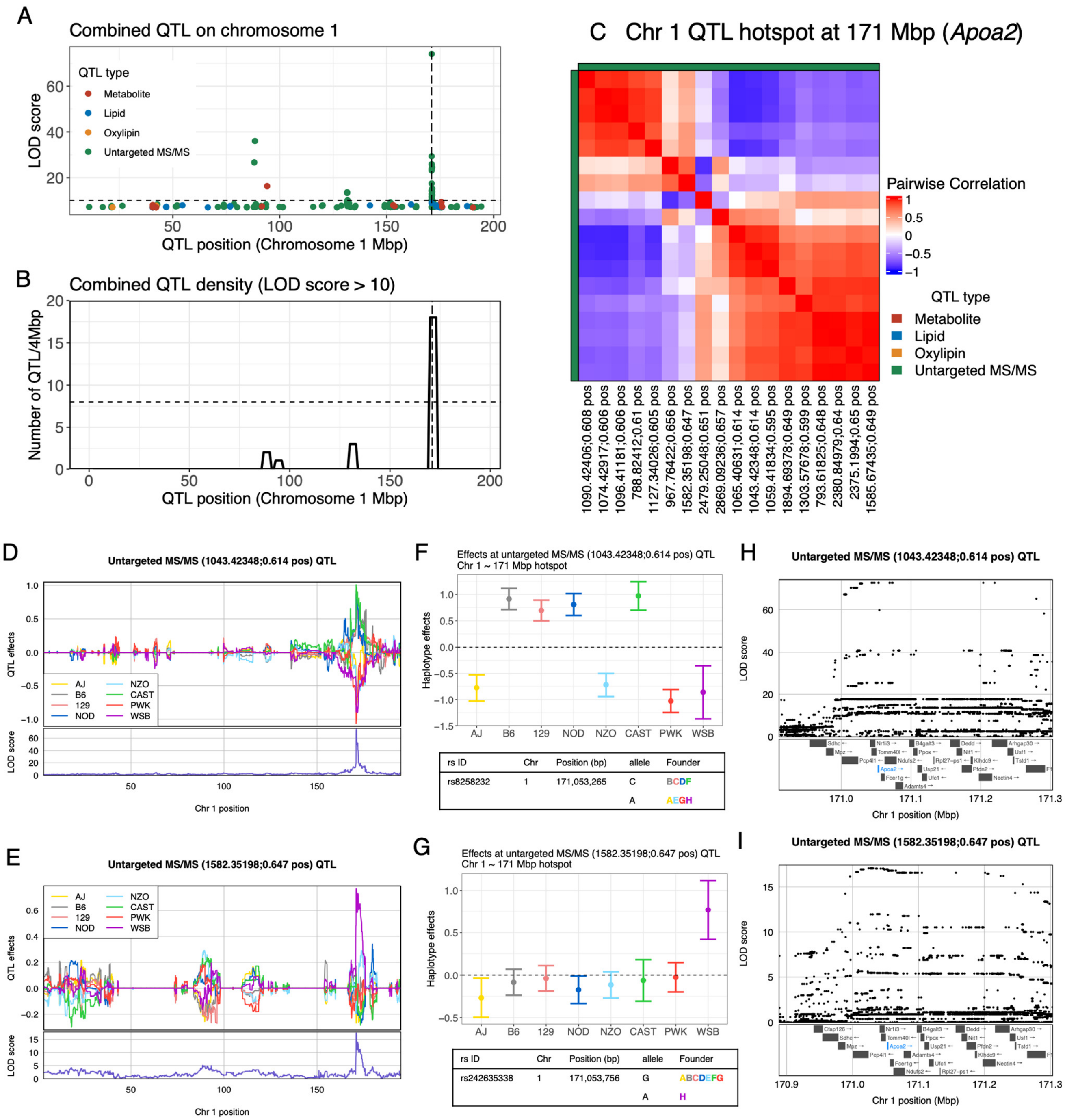

Figure D. Untargeted MS/MS QTL hotspot on chromosome 1 match known polymorphic peptides of APOA2, related to Figure 6. (A) Manhattan plot of detected QTL (LOD score > 7) on chromosome 1. Vertical dashed lines indicate the QTL hotspot. Horizontal dashed line represents a LOD score threshold of 10, used to define QTL hotspots. (B) The QTL density (LOD score > 10) across chromosome 1 combining data layers. The vertical dashed line represents the QTL hotspot. The horizontal dashed line represents a LOD score threshold of 10, used to define QTL hotspots. (C) The heatmap of the correlation matrix of haplotype effects for the QTL mapping to the hotspot. Aligned haplotype effects and genome scan on chromosome 1 for (D) 1043.42348;0.614 pos and (E) 1582.35198;0.647 pos. Haplotype effects with standard error bars at peak QTL locus for (F) 1043.42348;0.614 pos and (G) 1582.35198;0.647 pos. These haplotype effects match the allele distribution pattern of the missense variants rs8258232 and rs242635338, respectively. SNP association and genes within

the QTL 1.5 LOD drop support interval for (H) 1043.42348;0.614 pos and (I) 1582.35198;0.647 pos. Candidate gene of interest highlighted in blue. Annotations for gene models were removed for clarity.

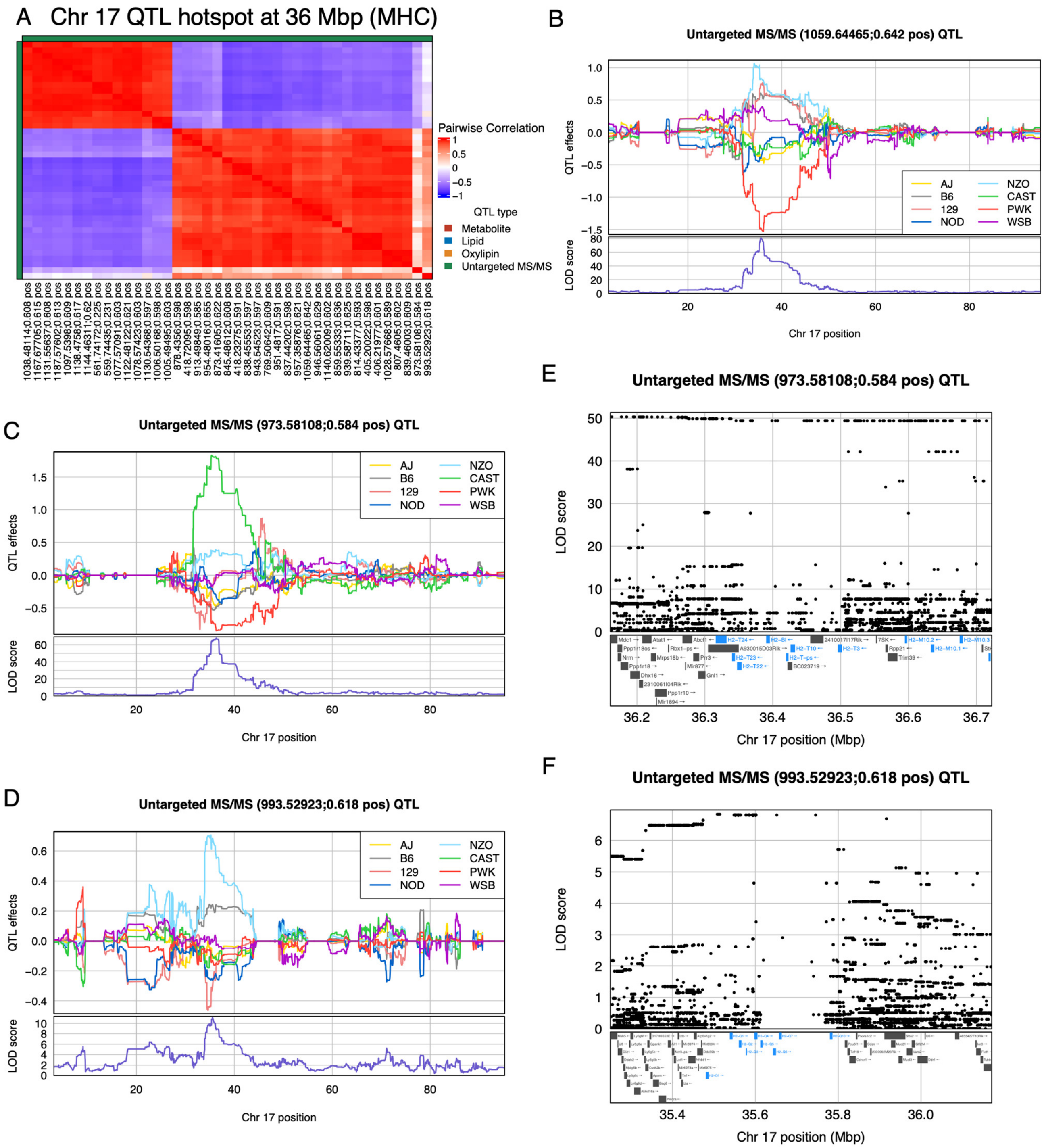

Figure E. Coding variation at major histocompatibility complex (MHC) revealed by untargeted MS/MS QTL hotspot, related to Figure 6. (A) The heatmap of the correlation matrix of haplotype effects for the QTL mapping to the hotspot at the MHC. Aligned haplotype effects and genome scan on chromosome 1 for (B) 1059.64465;0.642 pos, (C) 973.58108;0.584 pos, and (D) 993.52923;0.618 pos. SNP association and genes within the QTL 1.5 LOD drop support interval for (E) 973.58108;0.584 pos and (F) 993.52923;0.618 pos. Candidate gene of interest highlighted in blue. Annotations for gene models were removed for clarity.

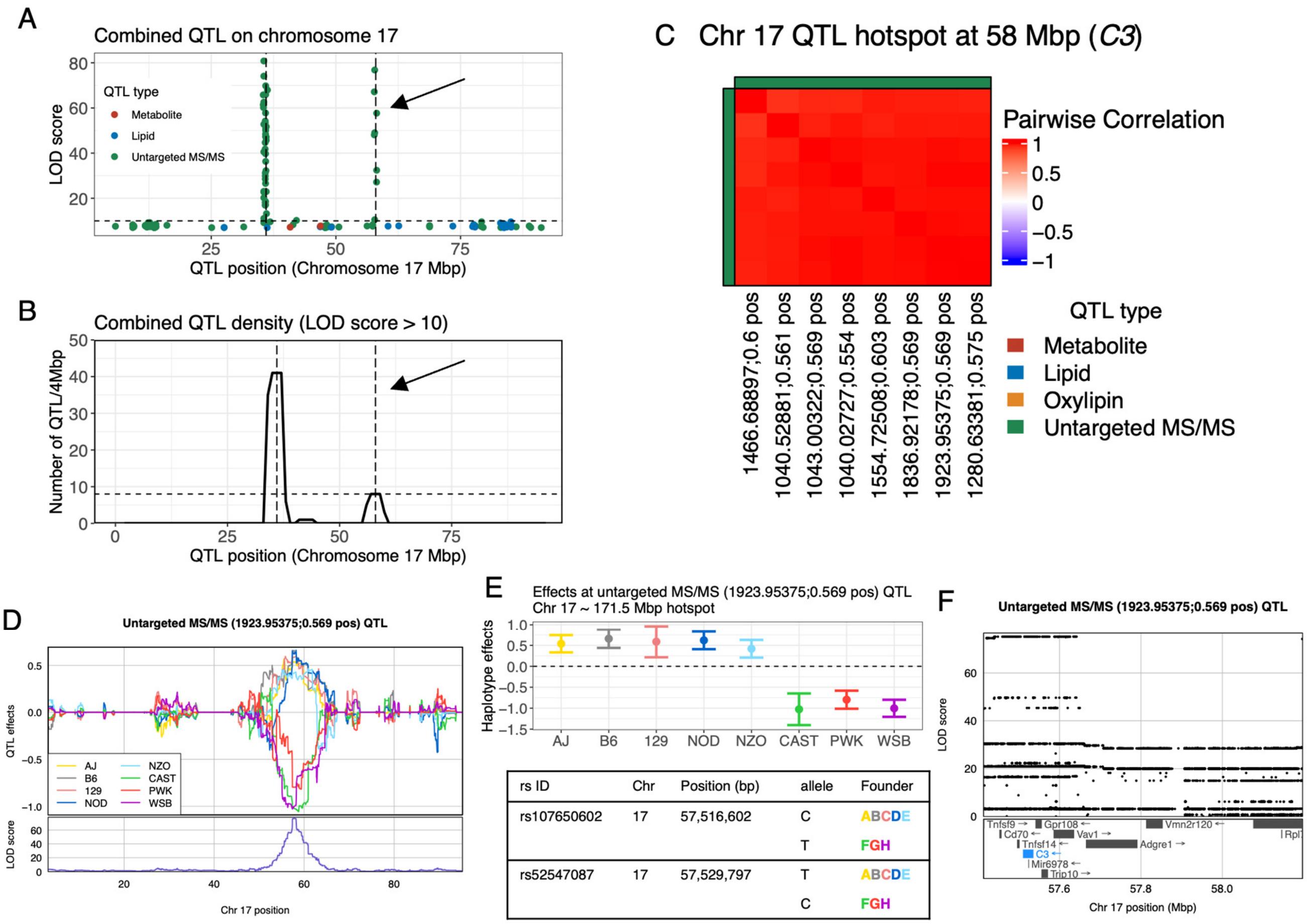

Figure F. Untargeted MS/MS QTL hotspot on chromosome 17 match known polymorphic peptides of C3, related to Figure 6. (A) Manhattan plot of detected QTL (LOD score > 7) on chromosome 17. Vertical dashed lines indicate the QTL hotspots. Horizontal dashed line represents a LOD score threshold of 10, used to define QTL hotspots. Black arrow highlights QTL hotspot at 36 Mbp. (B) The QTL density (LOD score > 10) across chromosome 17 combining data layers. The vertical dashed lines represent the QTL hotspots. The horizontal dashed line represents a LOD score threshold of 10, used to define QTL hotspots. Black arrow highlights QTL hotspot at 58 Mbp. (C) The heatmap of the correlation matrix of haplotype effects for the QTL mapping to the hotspot. For 1923.95375;0.569 pos, (D) aligned haplotype effects and genome scan on chromosome 17, (E) haplotype effects with standard error bars at peak QTL, and (F) SNP association and genes within the QTL 1.5 LOD drop support interval. These haplotype effects match the allele distribution pattern of the missense variants rs107650602 and rs52547087, respectively. Candidate gene of interest highlighted in blue. Annotations for gene models were removed for clarity.

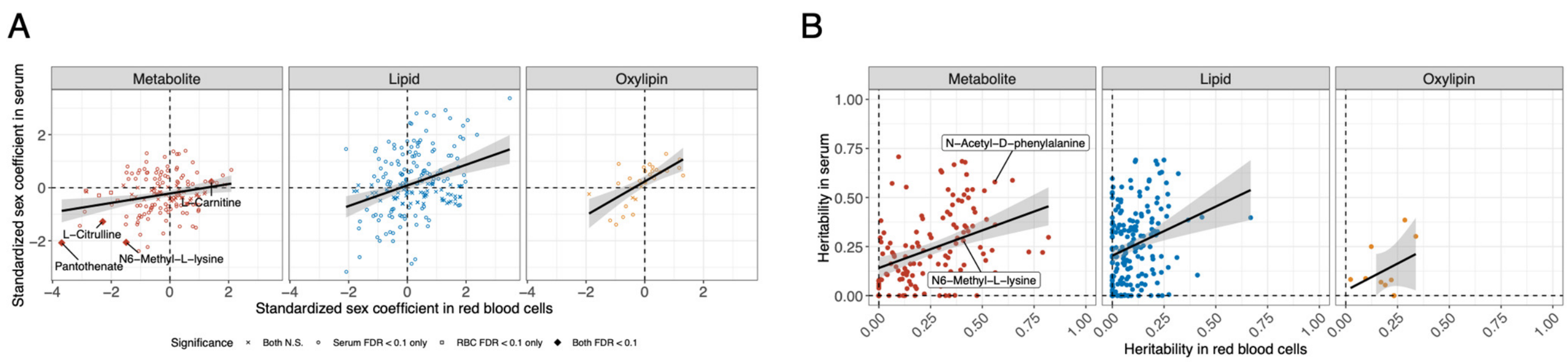

Figure G. Comparison of sex differences and heritability between serum and red blood cells (RBCs), related to Figure 7. Scatter plots of (A) sex differences and (B) narrow sense heritability, comparing metabolites, lipids, and oxylipins in serum to levels in RBCs. Best fit lines included with 95% confidence bands. Horizontal and Vertical dashed lines at 0 included for reference. Metabolites that have significant sex differences in both serum and RBCs are highlighted with labels. Heritability for N6-Methyl-L-lysine and N-Acetyl-D-phenylalanine are highlighted with labels, which map consistent QTL in serum and RBCs.
