# Supplementary material for: Genetic architecture of the murine serum metabolome reveals carboxyl esterases as master regulators of circulating fatty acid metabolism
Source: PLoS Genet. 2026 Nov 2;22(11):e1012223. doi: 10.1371/journal.pgen.1012223 (PMC13750656; doi:10.1371/journal.pgen.1012223)
Supplement: Supplementary file 2 [file pgen.1012223.s007.docx]

**Response to Reviewer Comments for PGENETICS-D-26-00636**

**Reviewer #1 Summary:** Keele et al. have used MS2 to generate large metabolomic, lipidomic, and oxylipin datasets in serum samples from a large and diverse cohort of 541 DO mice, and they have performed some genetic mapping on the resulting datasets. They have just under 1000 identified molecules, and a large set of 4500 unannotated/unidentified features. Many of these unannotated/unidentified features have significant differences across the cohort, so are presumably largely real metabolites/lipids, rather than noise or extremely-ephemeral intermediates. In general heritability of measurements is quite low (around 20%) for mouse cohorts, although certainly sufficient for this study given the sample size and high level of diversity. Interestingly (surprisingly, to me) many of the unannotated features _appear_ to be small peptides, or modified amino acids. There are also a handful of QTL hotspots, which is rather surprising to me that so many metabolites/lipids map to the exact same locus, and at such striking LOD scores.

**Authors’ reply:** We appreciate Reviewer #1’s accurate summary of our study and interest in some of its more intriguing results.

**Reviewer #1 Comment 1:** You mention that aligning these results with other omics data in Do mice would be extremely useful to identify candidates of interest. For the mice in this study, are there any previous studies on the these exact same mice? Or are e.g. the liver datasets you mentioned both from completely separate DO cohorts? It looks like they are all from separate cohorts, meaning the animals you took plasma from here have never been analyzed before? I see now ref 22, that’s the same mice, but only trait data (it seems). Granted of course, for plasma analysis, it is difficult to overlay with tissue data, since – as mentioned – often no who knows what tissue the metabolite came from, or even if they do, what percentage of it came from which tissues, and how persistent it is.

**Authors’ reply:** We apologize for any confusion. This specific cohort of DO mice has primarily been used for profiling metabolites/lipids/oxylipins (D’Alessandro et al. 2025 [ref 20], Nemkov et al. 2024 [ref 22], Nemkov et al. 2024 [ref 23]) and proteins/peptides/PTMs (Keele et al. 2026 [ref 67]) **in fresh and stored red blood cells (RBCs)**. The present study is the first and only one to focus on serum in this exact same cohort.

Additionally, they have been profiled for various blood antigen response phenotypes (e.g., Keele et al. 2025; PMC11532708), many of which are still in preparation for publication. Nor do the pQTL data from RBCs help refine candidates of interest because no local pQTL in RBCs mapped to the serum QTL hotspots. We have made this clearer up front in the **RESULTS**, whereas currently it is buried deeper in the comparison to RBCs section of the **RESULTS** and the **METHODS**.

We also noticed there are currently duplications in the **REFERENCES**: Nemkov et al. 2024 [ref 22] is duplicated [ref 27], Nemkov et al. 2024 [ref 23] is the pre-print version and should be replaced with ref 28, which is also duplicated [ref 59].

For candidate gene prioritization at the serum QTL hotspots via eQTL and pQTL, we leveraged data from essentially three other DO mouse cohorts (**Figure S2:** Svenson [liver], Attie [liver, adipose, islet, muscle, and heart], and Aging [heart, kidney, and bone]). We agree with Reviewer #1’s assessment that it is challenging in the context of metabolite/lipid/oxylipin QTL detected in serum/plasma. Even in contexts where the appropriate tissue is more obvious, these types of analyses (e.g., transcriptome wide analysis [TWAS]) are challenging and prone to false positives, which we emphasized in the **DISCUSSION**. Despite these challenges, we believe this approach proved valuable, providing some support for *Ces1b*, *Ces1c* (for TUDCA), and *Ces2h* at the QTL hotspots.

**Reviewer #1 Comment 2:** In Methods – and I also looked at your 2024 Blood paper – I don’t see it mentioned how the animals were controlled for their fast/fed state. Bile acids like TUDCA are going to radically change depending on the time since last meal, to the extent that it can be quite a challenge to get all mice on sufficiently-close feeding times such that its measurements can be particularly reliable, e.g. by timed fasting-refeeding. I don’t see it mentioned it in either paper, looking for keywords like “fast[ing]” and “fed” and from flipping through the methods.

**Authors’ reply:** We agree that ad libitum feeding does not standardize the interval since last food intake. The DO mice had ad libitum access to food and were not subjected to a timed fasting/refeeding protocol; the interval from last food access to blood collection was not controlled. Although serum collection followed a standardized procedure, feeding state remains an uncontrolled source of variation, particularly for bile acids. We have stated this explicitly in the Methods and added it as a limitation, tempering the interpretation of sex-associated bile-acid differences given the partial confounding of sex with shipment sub-cohort. In separate, unpublished experiments comparing B6 and FVB males and females under controlled feeding (see below), we also observe strain-by-sex differences in bile-acid profiles. This supports the biological plausibility of such interactions but does not establish feeding status or resolve sex-confounding within this DO cohort.

**Reviewer #1 Comment 3:** I certainly trust your QTL analyses, but I am still surprised at e.g. Fig 3A – that’s a huge number of lipids that map to chr8, like nearly all of them, and with massive LOD scores. Sure, the CES1 locus is important, but at those LOD scores and with that many lipids, it makes it seem like it is the master regulator of a huge range of lipids.

**Authors’ reply:** Thank you. We agree that Ces1 and Ces2 are prominent QTL hotspots, particularly Ces1 for lipids. Their prominence motivated their emphasis in the original title. Relatedly, in response to Reviewer #2’s Comment 5, this is why we favor retaining this title because it captures a central finding of the study, highlighting these dominant loci within a broader genetic map; it does not assign every measured feature to carboxylesterases. QTL hotspots can arise when an enzyme acts upstream in a shared pathway, causing related downstream features to map together. We also observe a Ces2 signal for liver and plasma lipid QTL in a separate DO cohort (**Figure S1C–D**); differences in mass-spectrometry protocols and feature-identification procedures may explain why that study does not show an equally dense hotspot.

**Reviewer #1 Comment 4:** Similar question for Fig 5A – that’s really a lot of metabolites mapping to the same locus with hugely-significant LOD scores, and Figure 6B, and Figure 7A. For 6B, the rationale that maybe it is MHC-related peptide fragments sounds reasonable to me, but the other hotspots are exceptionally ‘hot’. I don’t remember really ever seeing mQTL hotspots that hit such a large range of lipids with such large effect sizes. Normally what I expect is if you have, say, a knockout of a BCAA metabolizing enzyme, you see very striking buildups of a few particular metabolites, but then it eventually gets averaged out as you get further away from the key enzyme. What is the percent variance explained for e.g. LPC(22:6) in Figure 3C by the Cast allele? The effect is so large that I would expect e.g. a congenic line with just that region of Chr8 crossed into any of the other backgrounds to have just a massive impact on the levels of nearly all of those metabolites, even regardless of interacting genes – given anyway this is in DO mice. Are there any mice that are homozygous for Cast (or PWK or any of the other highly-striking alleles), and if so, do they have even more amplified effects? Or are there no such homozygotes, as maybe it would be embryonic lethal if so? (Can DOs be homozygotic at certain alleles? I don’t recall the breeding schema as to whether or not this is possible.)
**Authors’ reply:** Thank you; we agree that these hotspots merit an effect-size context, though there are some caveats to keep in mind. The QTL mapping is based on phenotypes that have been variance-stabilized and converted to normal quantiles to reduce the influence of outliers. LPC(22:6) maps to the chromosome 8 Ces1 region at 94.12 Mb with a LOD score of 34.49. The narrow-sense heritability of LPC(22:6) was estimated to be 69.1% (available in the **Data S1** file), and the QTL at 94.12 Mb on chromosome 8 had an R2-based effect size estimate of 53.1%. This proportion of variance estimate is for the additive multi-allelic locus term, after adjusting for covariates (i.e., sex), not the variance explained by CAST alone. This is because the QTL model we used tests founder-haplotype effects jointly, thus the LOD score and variance explained are not a single-allele effect. We have clarified that the hotspot pattern is compatible with a strong locus influencing multiple related features but does not by itself establish a universal “master regulator” or predict the outcome of a congenic cross. Homozygosity at loci can occur in DO mice, although the population is highly heterozygous (Svenson et al., 2012). Given Reviewer #1’s interest in the LPC(22:6) QTL on chromosome 8, here we have now plotted a view of the phenotype data stratified by the genotype at the locus:

The left most boxplot represents data from all the mice, thus capturing the overall phenotype distribution. For each founder allele, we generate a boxplot with both heterozygotes (medium circles) and homozygotes (large circles). A heterozygous mouse is thus present in two allele boxplots, whereas a homozygous mouse is in only one. One caveat is that we are inferring the most likely founder contributions for each mouse, given that there is uncertainty in haplotype inference. By comparing the medians of the allele boxplots to the overall mean (thick dashed line around 0), these largely reflect the relative orderings observed in **Figure 3C** (e.g., CAST is low, B6 and PWK are high).

There are 5 mice that are likely CAST/CAST at the QTL,13 that are likely PWK/PWK, and 2 that are likely B6/B6. Based on this, we can say homozygosity locus does not appear to be embryonically lethal. We note that this kind of dissection of the data is not feasible on a large scale in the context of a publication, given that it can be overwhelming. But it does highlight the kind of questions that can be further explored with the data in the format that we’ve made available.

**Reviewer #1 Comment 5:** You show the heritability, but it would be nice to know how much variation is due to residuals, how much due to sex, etc. I guess this is already in your data (and maybe is in a supplemental table somewhere).
**Authors’ reply:** Thank you. The **Data S1** file includes feature-level narrow-sense heritability estimates, along with sex-effect coefficients, standard errors, p-values, and FDR-adjusted p-values. It does not partition total variation into separate shares for genetics, sex, plate/batch, PC1, and residual error, though it would not be hard for researchers to estimate these types of quantities themselves, such as sex effect size or QTL effect size) using the processed data that we have also made available (<https://doi.org/10.6084/m9.figshare.31286470> or <https://churchilllab.jax.org/qtlviewer/Zimring>). We have refrained from including such summary statistics with an eye towards clarity and the added burden of describing additional statistical methods. Furthermore, given our caution against over-interpreting the sex coefficients due to its partial confounding, we focused on larger trends across multiple features rather than specific variance explained estimates.

In terms of the actual mixed model, sex is a fixed effect, and sample plate and PC1 are covariates where specified; heritability is estimated from the additive-genetic and residual variance components. We have clarified in the **Methods** and **Data S1** caption that these summaries are not a complete variance decomposition. In particular, the complement of h² is not a full partition of raw phenotype variance into these sources.

**Reviewer #1 Comment 6:** Is there a data table that just has the abundances of all of the features in each of the mice? I see on your lab website things like the “dataset.lipids.stored.rds” but I’m not 100% sure this is the file I’m looking for. “dataset.metabolites.serum.rds” is that the other one I want? I downloaded it – it looks like probably yes. If that’s right, then that looks sufficiently easy to follow along.
**Authors’ reply:** Yes. As Reviewer #1 notes, we have made the individual-mouse data matrices available as R binary files (e.g., dataset.metabolites.serum.rds), either from <https://doi.org/10.6084/m9.figshare.31286470> or <https://churchilllab.jax.org/qtlviewer/Zimring>. There are analogous files for lipids, oxylipins, and untargeted features. If one of those files is loaded into R, such as by this code example:

```
# Read in data file
dataset.metabolites.serum <- readRDS(“local_path/dataset.metabolites.serum.rds”)
# Extract data matrix
mouse_matrix <- dataset.metabolites.serum$data$rz

# dataset.metabolites.serum$data$rz is the fully-transformed normal quantiles data used for QTL mapping
# dataset.metabolites.serum$data$norm is the variance-stabilized data (prior to normal quantile transformation)
# dataset.metabolites.serum$data$raw is the untransformed data
```

Code generating all the figures is also included in the above figshare repository, and could be used to understand the data structures and further explore the data. **Data S1** is the feature-level summary of sex effects and heritability, rather than the per-mouse matrix.

**Reviewer #1 Comment 7:** It would be nice to see a few more positive controls of expected outcomes – like do you expect kynurenine to go up in females? Is there a good metabolite in here that you could show as a convincing proxy of fasted/fed state? On MOFA, can you start to guess what factor 1-2-3-4 are? I assume one is genetics and one is residuals, probably another one is sex, but perhaps another one is fasted state? Is there any good way to figure that out? I see you have correlated them with the QTL hotspots, but within the list of features you measure, some may be characterized well enough to know “ah ha, this is a sex-linked metabolite”.
**Authors’ reply:** Thank you. We agree that examples tied to recognizable biology will help orient readers. Trimethylamine N-oxide and kynurenine were both female-associated in this cohort (**Data S1)**. We qualified these findings because sex was partially confounded with shipment sub-cohort, so they should be viewed as observed associations rather than robust independent positive controls. In terms of sex, our additional data for bile acids in response to Reviewer #1 Comment 2 does demonstrate elevated levels in females compared to males for B6, which is one of the founder strains of the DO. It is important to not over-interpret these comparisons given the differences in experimental context; neverthess they do provide some external support for consistency.

In terms of clear positive controls that support the quality of our data, we also pointed readers to the *Apoa2*-associated peptide-like features in **Figure S4**, whose founder-effect patterns match *Apoa2* coding variation. Because mice had ad libitum food access and were not on a timed fasting/refeeding schedule, we cannot designate an internal feature as a reliable fasted/fed control.

MOFA factors are latent summaries, and thus not directly labeled in terms of genetics, sex, feeding, or residual variation. Furthermore, our main goal with MOFA is to confirm the co-regulatory patterns of the QTL hotspots across the various data layers. For this reason, we actually filtered input data to features mapping to the QTL hotspots. Doing so allowed us to confirm that hotspot loci explained significant variation in MOFA factors, thus suggesting the latent factor likely represents the features at that mapping QTL to that hotspot, as well as their breakdown across the data layers. For example, in **Figure 2F**, we describe how the lipid-enriched factor 1 associated with the chromosome 8 *Ces1* hotspot and factor 6 associated with untargeted MS/MS features at the chromosome 17 hotspot. Factor 2 appears to represent metabolites mapping QTL to the chromosome 8 *Ces2* hotspot. Factor 3 seems to absorb variation from mostly metabolites and untargeted MS/MS at the chromosome 8 *Ces2* hotspot and chromosome 10 hotspot. Factor 4 is largely capturing lipid variation regulated by the chromosome 9 hotspot.

We have essentially focused this MOFA analysis on the hotspots intentionally. Researchers could take the data we have made available and explore its variation differently. For example, not filtering and looking for other biological or technical drivers of variation (strong individual QTL, sex, etc.). We note there can be a lot of nuance to running MOFA, such as adequate normalization across data layers and the relative sizes of the data layers – thus filtering down the data some was appealing for our analysis.

**Reviewer #1 Minor Comment 1:** On page 10, you have “though these approaches are still subject to assumptions and multiple sources of uncertainty (e.g.,) .” The parenthetical “e.g.” is missing the example.
**Authors’ reply:** Thank you for pointing out the missing examples. We completed the parenthetical with some examples: uncertainty in the founder-haplotype effect estimates and assumptions used to group haplotypes into allelic series.

**Reviewer #1 Concluding Statement:**
The authors have generated a large dataset in a diverse set of mice. I would like to know how the fasting/fed state was made consistent (again, perhaps I overlooked it in methods somewhere), particularly as I am surprised by how many features are mapping to the hot spots – the hot spots are blazing infernos both by their LOD score (especially for such a large cohort) and the number of features mapping to the same spot. This is a good (and massive) dataset and I agree with the analyses done. That said, given the surprise – to me – that so many metabolites map so significantly despite the (expected) moderate heritability, I think this paper would do better with a few more basic QC analyses at the start of the manuscript. For instance, surely by this point there are some metabolites that are already known to be elevated in PWK or CAST (or whatever) strain, and B6 certainly has many plasma features where it tends to be particularly low or particularly high. Similar as mentioned in comment #7, there must be some clear sex-linked metabolites (I guess you are showing these in Figure 1B). I see trimethylamineoxide is generally elevated in the plasma from women than from men. Seeing a list of examples like that, and those which are known to vary from strain (when such information is available) would help with interpreting the data.

But I think my only significant scientific concern is about the fasting of the mice, especially considering that males and females are batch-confounded, can you really be sure that e.g. TDCA is upregulated in females so dramatically, or whether it is that the cage changes or feeding or time or day or whatever was systematically slightly different in one cohort than the other? Fasting affects so many plasma metabolites so dramatically, but particularly the bile acids. Such huge QTL hotspots for such large ranges of metabolites make me wonder what alternate explanations could be, so I think showing additional basic examples of “this is an obesity-related metabolite, it is 2-fold up in mice that are > 40 g compared to those < 30g, voila.” would help quite a lot. Actually now that I think about it, I am kind of surprised that body weight is not used as a cofactor in any of these analyses – with such a large cohort of DOs there should be a pretty good range, and it should track closely with the levels of quite a few compounds.

In general I like this paper: I just would like clarification on the fast/fed state, and some additional ‘zoom in’ examples on specific metabolites that are known in literature, to serve as positive controls. I think that should be a pretty quick revision. Actually, I am now looking at the supplemental figures and Figure S4 is already along these lines. So… I guess I just want a bit more of that, and with your other key dependent and independent variables, somewhere around the end of Fig 1 or beginning of Fig 2.

**Authors’ reply:** We appreciate Reviewer #1’s enthusiasm and clear and thorough interest in our work. Unless the reviewer and the editors feel strongly about this point on moving **Figures S4** to further emphasize it, we kindly request to retain it in the supplement because it presents a detailed, nine-panel characterization of the chromosome 1 untargeted MS/MS QTL hotspot and the correspondence between founder-effect patterns and *Apoa2* missense-variant distributions. The linked panels document hotspot localization, founder-effect patterns, and variant support for a focused locus-specific analysis associated with Figure 6; **moving selected panels next to Figures 1–2 would separate that evidence and introduce a technical mechanistic analysis before the relevant QTL results**.

We have addressed the broader request by adding concise examples in the early Results: female-associated trimethylamine N-oxide and kynurenine, with the qualification that sex is partly confounded with shipment sub-cohort. Though not included in this manuscript, we share with Reviewer #1 (see response to comment #2) a separate, unpublished comparison of B6 and FVB males and females under controlled feeding that also showed strain-by-sex bile-acid differences; this supports biological plausibility but does not resolve the shipment confounding in the DO cohort.

We added an introductory note that the *Apoa2*-region peptide-like features provide a complementary example of genotype-associated MS/MS signals, with the detailed evidence retained in **Figure S4**. We also clarify that food was available ad libitum and time since last intake was not controlled, so the dataset cannot provide a reliable fasted/fed control. Body weight was not recorded; we now identify it as an unmeasured source of variation and explain why it was not included as a covariate. Despite this limitation, we do note that we still observe strong and highly varied QTL signal across the measured features, suggesting the lack of statistical adjustment for body weight is not greatly limiting our statistical power, nor is potential confounding of body weight with our features (e.g., metabolites) driving all signal to an underlying body weight QTL (given the varied QTL observed). We respectfully submit that these main-text examples provide the requested biological anchors while **Figure S4** remains with the detailed locus-specific analysis.

**Reviewer #2 Summary:** The manuscript by Keele and colleagues provides a detailed analysis of the genetic regulation of metabolite levels in DO mice. Using a large population of DO mice and an comprehensive array of metabolomic platforms, the authors define a large landscape of metabolism under genetic regulation. A critical finding is the existence of mQTL hotspots in which multiple metabolites are associated with a single locus. The authors then characterize the hotspot loci using a series of analysis that utilize an extensive array of expression (eQTL) and protein (pQTL) data publicly available, and the authors provide a detailed assessment of candidate genes at the 7 hotspots identified. Overall, it is well written and data is presented clearly. There are some suggestions to improve some clarity for the reader.
**Authors’ reply:** Thank you for the kind assessment and constructive criticism.

**Reviewer #2 Comment 1:** The authors have provided a significant amount of detail for the metabolomic assays and platforms. However, given the technical nature of metabolomics some additional information or edits would improve the clarity of the data generated. For example, the authors state in subsequent sentences: “Mass spectrometry for metabolites, Oxylipins, and lipids in red blood cells (RBCs) was described previously for this DO cohort [20,22,23]” and then “metabolomics extraction and analyses in 96 well-plate format were performed as described [56, 57]”. While the use of referenced methods is helpful, specific items such as mass spectrometry library size likely has increased over time and adding some additional information is helpful. For example, all 240 metabolites in table S1 have standards run as part of the process (from manuscript 58)? These are part of the single concentration standards used on each plate? A suggestion would be to expand or clarify some of the methods and if necessary, create a supplemental method section.

**Authors’ reply:** Thank you for pointing out this distinction. The 240 entries are metabolite signals annotated and integrated in El-Maven with our in-house standard library and are reported as median-normalized relative abundances. As described in the **Methods**, a blank containing the standard mixture in Ref. 58 was injected twice per plate, and the same quality-control sample was injected five times per plate. These injections monitor analytical performance; they are not analyte-specific calibration curves for every entry, and we do not report absolute concentrations. A standard library – now ~10,000 compounds – has also been characterized under the same analytical conditions and used as external reference for peak assignments. All of the above is now explicitly stated in the methods.

**Reviewer #2 Comment 2:** Having some statements relaying the overall confidence of the matches to libraries would also be helpful. MS/MS and spectral matches do have some caveats and relaying these in the manuscript is appropriate as the nuances of this data type maybe foreign to readers of PLOS Genetics.

**Authors’ reply:** Thank you. We stated the identification confidence and limitations by data layer. Metabolite peaks were manually assigned and integrated at sub 5 ppm error in El-Maven against an in-house standard library, while lipid spectra were searched with LipidSearch v5.0; these workflows do not provide one comparable confidence score across all annotations, so we avoided implying that every named feature is equally confirmed. For the untargeted peptide/protein search, only target-decoy/Percolator matches passing a 1% peptide-spectrum-match false discovery rate are designated high-confidence (70 high-confidence peptide-spectrum matches, 30 peptide entries, and 28 protein accessions). Other MS/MS features remain unmatched or ambiguous and are not assigned definitive identities.

**Reviewer #2 Comment 3:** What was the rationale to choose a 4Mb region to use for the hotspot analysis and why were 8 metabolites required to define a hotspot. Please include some rationale for this choice.
**Authors’ reply:** LOD > 10 is a stringent QTL filter, above the simulation-based LOD 8.3 genome-wide threshold, and thus ensures our hotspot analysis focuses on very high confidence QTL. The 4 Mb window and the minimum count threshold of eight QTL are pragmatic (and admittedly arbitrary) criteria for grouping nearby peaks and prioritizing large clusters. Also, the QTL count includes feature-locus associations across data layers, not only chemically identified metabolites. We also note that DO cohorts have higher levels of linkage disequilibrium than large natural populations (i.e., genome-wide association studies in human biobank data), thus we have used wider intervals, like 4 Mb windows, for defining hotspots or classifying eQTL or pQTL as local (i.e., *cis*) or distant (i.e., *trans*), as in the eQTL/pQTL gene prioritization analysis. See plot below for a sensitivity analysis (which can be performed using **Data S2**), where we also evaluated grouping windows of 2 Mb and 1 Mb, as well as minimum count thresholds of 6 and 10. With the 4 Mb window (reported results), changing the minimum count to 6 or 10 yielded 11 or 5 hotspots, respectively. A 2 Mb window produced the same results. A 1 Mb window and minimum count thresholds of 6, 8, and 10 yielded 8, 5, and 5 hotspots, respectively. These results reveal the hotspots to be fairly robust to these parameters.

More broadly, we did not mean to ascribe statistical significance to these parameters. Likely empirical approaches to estimating a statistical hotspot minimum count threshold, such as randomly sampling genomic positions for observed QTL to characterize a null distribution of overlapping QTL counts, would find hotspots smaller than 8 to be statistically significant, but would also represent an overwhelming number of results to report and investigate further. We do believe this highlights the value of these data as a resource, given smaller QTL hotspots could still yield new findings, particularly if the metabolites, lipids, or oxylipins have are of interest to a given researcher.

**Reviewer #2 Comment 4:** The analysis of MS/MS spectra for protein and peptide moieties is novel. The result is a bit difficult to understand in the context of MHC in the results section. Adding some additional verbiage specific to Chr 17 hotspot would be helpful. Further in the same section, the ApoA2 locus is discussed. The result is intriguing given its somewhat complex effects on atherosclerosis and lipids and perhaps pleotropic effects on metabolism. The authors have used haplotypes at the ApoA2 locus to contrast specific metabolites under genetic regulation. Describing briefly the biological implications of the ApoA2 results in the discussion may better demonstrate the value of the data and approach taken.
**Authors’ reply:** Thank you. We have clarified that the chromosome 17 36 Mb hotspot spans MHC class II and consists of untargeted MS/MS signals, many of which are peptide-like by ionization state (double/triple charge). Only a subset of all signals received high-confidence peptide-spectrum assignments based on y/b series ion annotations (Supplementary data 4), so the data support genetic association with circulating peptide-like features but do not establish that every feature is an MHC peptide or prove altered antigen presentation. We also expanded the *Apoa2* discussion: two QTL for peptide-like features that map to the chromosome 1 locus have founder-effect patterns matching the strain distribution of distinct Apoa2 missense variants. This illustrates how circulating MS/MS can reveal genotype-linked peptide variation and motivates *Apoa2* as a candidate relevant to lipoprotein biology, while not demonstrating altered ApoA-II abundance or a causal effect on lipid or atherosclerosis phenotypes. It also represents a strong true positive signal demonstrating the quality for the MS/MS data, this cohort’s genotype data, and the reference genotypes for the founder strains.

**Reviewer #2 Comment 5:** The title mentions carboxyl esterases as master regulators of circulating fatty acid metabolism, but this is only partially discussed in the manuscript. The title should be revised to better reflect the broad nature of the loci discussed. The manuscript contains several vignettes of data, and each are interesting but not solely related to carboxyl esterases nor fatty acids.

**Authors’ reply:** Thank you for this suggestion, which highlights a challenge underlying writing papers for large-scale genomic screens of molecule (i.e., omic) phenotypes. As is often the case with these types of studies, the study maps loci beyond one or two loci, such as *Ces1* and *Ces2,* and in this case, covers metabolites, lipids, oxylipins, and peptide-like features. Unless the reviewer and the editors feel strongly otherwise, we respectfully propose retaining the original title, “Genetic architecture of the murine serum metabolome reveals carboxyl esterases as master regulators of circulating fatty acid metabolism,” because it highlights the prominent *Ces1/Ces2* lipid QTL hotspots revealed by the study, which do stand out prominently (see QTL densities in **Figure 2** or in the response to Review #2’s Comment 3). In response to Reviewer #1’s comment 4 above, we provide additional effect-size context for the *Ces1* association, including the multi-founder locus effect for LPC(22:6) (see also response to Reviewer #1’s Comment 4). The **Abstract** and **Results** also describe the full scope of the work: 1,933 mQTL across 240 metabolites, 561 lipids, 43 oxylipins, and 4,465 MS/MS features, including additional loci associated with peptide-like features and other metabolic pathways. We therefore favor the original title alongside this broader description of the study.

**Reviewer #2 Comment 6:** For their figure 3, they're characterizing QTL allelic founder effects (tri-allelic, bi-alleleic) based off visual inspection of R/qtl2 BLUP plots. Are the authors are relying on collapsed haplotype effects (figure 3D, G, J) and identifying patterns of non-overlapping haplotype effect signal. The authors have created an alternative method to characterize alleles at a locus (TIMBR). Would use of this analytical method improve linkages between alleles and metabolites (similar to ApoA2 above)? Also, the last sentence of 2nd paragraph on page 4 is missing information- though these approaches are still subject to assumptions and multiple sources of uncertainty (e.g., ). What is missing after e.g.?
**Authors’ reply:** In terms of any reported statistics or figures, we did not collapse the eight founder haplotypes. The relevant figures show the full eight-founder haplotype BLUPs; “bi-allelic” and “tri-allelic” are qualitative descriptions of their visual patterns, not formal groups used in QTL testing. This is also the case with Apoa2, where again based on visual inspection, the haplotype effects clearly match the allele-to-founder patterns expected for specific SNPs.

We also want to note that none of the authors of this work were responsible for the TIMBR method, though it did come out of the PhD lab where the lead author trained, thus he is highly familiar with it. Rather than modeling eight alleles as is the convention for QTL mapping in the DO, TIMBR is a fully Bayesian model of the allelic series at a QTL and can thus provide formal posterior inference on the allelic series. This can address how functional alleles are distributed across the founder strains, but does not directly attempt to identify causal variants or mechanisms by itself. Given Reviewer #2’s interest in TIMBR, we have run it for three QTL: the two QTL for untargeted MS/MS features that map to Apoa2 and the QTL for the lipid LPC(22:6) that maps to Ces1. The circular plots below represent the allelic series of each QTL, with the vectors connecting founder strains (coded as letters) representing the posterior probability mass that two founder strains share a functional allele. For all three QTL, TIMBR supports our visual assessment of allelic series patterns: bi-allelic for the two QTL that map to Apoa2 and tri-allelic for the QTL mapping to Ces1. In the case of the untargeted MS/MS features, this nicely matches the known genetic variation patterns and thus can provide further support for specific causal variants.

These examples also highlight the challenge of formally modeling the allelic series; for example, the tri-allelic series with maximum posterior probability for the LPC(22:6) QTL is still just 0.4515, suggesting truly resolving the functional allelic series is subject to a high level of uncertainty. Furthermore, TIMBR is a fully Bayesian approach, and is thus subject to prior specification. For this reason, we have not run TIMBR systematically for all QTL, as its interpretation can be nuanced and more easily managed for a handful of QTL of interests.

In terms of the paper, we have made the descriptive nature of these labels explicit, soften categorical wording, and complete the example in the Discussion: “uncertainty in founder haplotype effect estimates and assumptions used to group haplotypes into functional allelic series”.

**Reviewer #2 Comment 7:** In the methods (Sex differences) the authors mention " As mentioned above, sex is partially confounded with batch because animals were shipped in six separate sex-specific sub-cohorts.” The analysis of sexual dimorphism indicates 50% of the metabolites (~119) and 52% of lipids (~2897) were sex biased. The authors then state 14 of 15 metabolites and 26 of 29 lipid species were elevated levels in males. This statement seems to contradict the above. Are the authors referring to column G of the S1 table (pathway)? If so, revising this sentence to reflect this annotation is needed.
**Authors’ reply:** Thank you for catching this ambiguity. The 14/15 and 26/29 counts refer only to the fatty-acid-pathway subset in Data S1 with significant sex differences, not to all sex-biased metabolites and lipids. We revised the sentence to read: “Among fatty-acid-pathway features with significant sex differences, 14 of 15 metabolites and 26 of 29 lipid species had higher levels in males.”

**Reviewer #2 Comment 8:** The authors note that although they adjust for batch and use a stringent significance threshold, it is possible that confounding could still induce false positive sex differences. The authors should clarify how many of the mQTL identified are from metabolites that differ between sexes. Is there any concern that mQTL identified are sex specific? Most importantly are the mQTL identified at the hotspots robust to the confounding of batch and sex. This seems to be a limited number of mQTL so could a sex stratified analysis confirm the QTLs exist in both sexes even at a relaxed threshold?
**Authors’ reply:** Using the FDR < 0.01 sex-effect results in **Data S1** and the LOD > 7 QTL in **Data S2**, 83 of 163 metabolite QTL (covering 62 distinct metabolites) involve metabolites with significant sex effects. At the stricter LOD > 10 threshold, 18 of 51 metabolite QTL (16 distinct metabolites) overlap sex-biased metabolites. These are overlap counts, not evidence of a genotype-by-sex interaction. All combined QTL scans include sex and sample-plate covariates, as well as PC1 cluster for metabolites, oxylipins, and untargeted features (PC1 was omitted for lipids). Furthermore, population structure or imbalanced genetic relatedness are accounted for with a leave-one-chromosome-out kinship term. In terms of identifying QTL that are independent of sex, which is what we have attempted, we do not believe the confounding of sex and batch should be an issue, since there is no reason to believe genotype at loci are confounded with sub-cohort batches. Including these covariates should reduce variation independent of genotype in the QTL scans, but it is impossible to fully disentangle sex from the sex-specific shipment sub-cohorts. Given the sex confounding issue (as well as roughly halving the sample size), we have not performed, nor would we recommend, sex-stratified QTL scans, so we cannot claim that each hotspot was independently detected in both sexes. We have stated this limitation, report the overlap counts, and avoid implying sex-specific or sex-independent effects.

**Reviewer #2 Comment 9:** Their serum-to-RBC QTL overlap analysis does indicate tissue or cell type specificity of metabolism (and mQTL). This is an important point but the lack of mitochondria in RBC could explain some of these differences. Arguably, it would be better to compare different tissues or cells with similar organelles than the complex mixture of serum and RBC’s?

**Authors’ reply:** We agree that serum and mature red blood cells are not equivalent tissues: serum is a composite circulating matrix, whereas mature red blood cells lack mitochondria and represent an intracellular compartment. We compared serum with previously published red blood cell measurements from the same DO cohort, although the sample overlap was incomplete, to ask whether genetic associations are shared across these blood compartments, not to treat them as organelle-matched tissues or infer tissue of origin. We replaced “cell-type-specific” with “matrix-specific” or “compartment-specific” where appropriate and note that these biological differences limit interpretation; comparisons among tissues or cell types with more similar organelle content would be a useful next step.

**Have all data underlying the figures and results presented in the manuscript been provided?**
Large-scale datasets should be made available via a public repository as described in the *PLOS Genetics* [data availability policy](https://nam04.safelinks.protection.outlook.com/?url=http%3A%2F%2Ftrack.editorialmanager.com%2FCL0%2Fhttp%3A%252F%252Fjournals.plos.org%252Fplosgenetics%252Fs%252Fdata-availability%2F1%2F010f01a05afaaa99-e8fea31b-b232-4c83-8a3b-a0ccfd9c167f-000000%2FDmeJDgsxttAMAeUuim2qDUE0tk99aiIQYDBP_J2FpzA%3D258&data=05%7C02%7Cgkeele%40rti.org%7C7d3cc1a40c4542ffa4d708df07d7d7ba%7C2ffc2ede4d4449948082487341fa43fb%7C0%7C0%7C639238295775989959%7CUnknown%7CTWFpbGZsb3d8eyJFbXB0eU1hcGkiOnRydWUsIlYiOiIwLjAuMDAwMCIsIlAiOiJXaW4zMiIsIkFOIjoiTWFpbCIsIldUIjoyfQ%3D%3D%7C0%7C%7C%7C&sdata=UK2jOvTbzqR8FY9y7B5D5ULedr4tEdAMw6GFT9hnY4w%3D&reserved=0), and numerical data that underlies graphs or summary statistics should be provided in spreadsheet form as supporting information.

The data are publicly available through an open access, interactive QTL viewer repository at [**https://churchilllab.jax.org/qtlviewer/Zimring**](https://churchilllab.jax.org/qtlviewer/Zimring)

Reviewer #1: Yes

Reviewer #2: Yes
