# Supplementary material for: Genetic architecture of the murine serum metabolome reveals carboxyl esterases as master regulators of circulating fatty acid metabolism
Source: PLoS Genet. 2026 Nov 2;22(11):e1012223. doi: 10.1371/journal.pgen.1012223 (PMC13750656; doi:10.1371/journal.pgen.1012223)
Supplement: Peer review history

# Author comment: Author response to Decision Letter 0

**DOI:** 10.1371/journal.pgen.1012223.r001

**Transfer Alert**

This paper was transferred from another journal. As a result, its full editorial history (including decision letters, peer reviews and author responses) may not be present.

21 Jun 2026

# Peer review: Decision Letter 0

**DOI:** 10.1371/journal.pgen.1012223.r002

**Authors:** Zoltán Kutalik, Ana Viñuela

31 Aug 2026

PGENETICS-D-26-00636

Genetic architecture of the murine serum metabolome reveals carboxyl esterases as master regulators of circulating fatty acid metabolism

PLOS Genetics

Dear Dr. D'Alessandro,

Thank you for submitting your manuscript to PLOS Genetics. After careful consideration, we feel that it has merit but does not fully meet PLOS Genetics's publication criteria as it currently stands. Therefore, we invite you to submit a revised version of the manuscript that addresses the points raised during the review process.

Please submit your revised manuscript within by Sep 30 2026 11:59PM. If you will need more time than this to complete your revisions, please reply to this message or contact the journal office at plosgenetics@plos.org. When you're ready to submit your revision, log on to https://www.editorialmanager.com/pgenetics/ and select the 'Submissions Needing Revision' folder to locate your manuscript file.

Please include the following items when submitting your revised manuscript:

* A letter that responds to each point raised by the editor and reviewer(s). You should upload this letter as a separate file labeled 'Response to Reviewers'. This file does not need to include responses to formatting updates and technical items listed in the 'Journal Requirements' section below.

* A marked-up copy of your manuscript that highlights changes made to the original version. You should upload this as a separate file labeled 'Revised Manuscript with Track Changes'.

* An unmarked version of your revised paper without tracked changes. You should upload this as a separate file labeled 'Manuscript'.

If you would like to make changes to your financial disclosure, competing interests statement, or data availability statement, please make these updates within the submission form at the time of resubmission. Guidelines for resubmitting your figure files are available below the reviewer comments at the end of this letter.

As the corresponding author, your ORCID iD is verified in the submission system and will appear in the published article. PLOS supports the use of ORCID, and we encourage all coauthors to register for an ORCID iD and use it as well. Please encourage your coauthors to verify their ORCID iD within the submission system before final acceptance, as unverified ORCID iDs will not appear in the published article. Only the individual author can complete the verification step; PLOS staff cannot verify ORCID iDs on behalf of authors.

We look forward to receiving your revised manuscript.

Kind regards,

Ana Viñuela

Academic Editor

PLOS Genetics

Zoltán Kutalik

Section Editor

PLOS Genetics

Aimée Dudley

Editor-in-Chief

PLOS Genetics

Anne Goriely

Editor-in-Chief

PLOS Genetics

**Additional Editor Comments:**

Among the points raised by the reviewers, we consider the following to be the most critical and requiring resolution to the satisfaction of the reviewers:

Methodological clarity: Please address the reviewers' questions regarding the feeding/fasting state of the mice, quality control of metabolites, use of spectral matches, and the covariates included in the analyses. All information essential to understanding the methodology should be presented directly in the manuscript, rather than referring readers to additional publications.

Genetic analyses and sex differences: Additional clarity is needed here as well. In particular, we would appreciate efforts to make this section more accessible to a general reader.

Thank you for the opportunity to review your work.

Respectfully yours,

Ana

**Journal Requirements:**

If the reviewer comments include a recommendation to cite specific previously published works, please review and evaluate these publications to determine whether they are relevant and should be cited. There is no requirement to cite these works unless the editor has indicated otherwise.

1) Please provide an Author Summary. This should appear in your manuscript between the Abstract (if applicable) and the Introduction, and should be 150-200 words long. The aim should be to make your findings accessible to a wide audience that includes both scientists and non-scientists. Sample summaries can be found on our website under Submission Guidelines:

https://journals.plos.org/plosgenetics/s/submission-guidelines#loc-parts-of-a-submission

2) Please upload all main figures as separate Figure files in .tif or .eps format. For more information about how to convert and format your figure files please see our guidelines:

https://journals.plos.org/plosgenetics/s/figures

3) We have noticed that you have uploaded Supporting Information files, but you have not included a complete list of legends. Please add a full list of legends for your Supporting Information files after the references list.

4) Please amend your detailed Financial Disclosure statement. This is published with the article. It must therefore be completed in full sentences and contain the exact wording you wish to be published.

i) State what role the funders took in the study. If the funders had no role in your study, please state: "The funders had no role in study design, data collection and analysis, decision to publish, or preparation of the manuscript."

ii) If any authors received a salary from any of your funders, please state which authors and which funders..

If you did not receive any funding for this study, please simply state: u201cThe authors received no specific funding for this work.u201d

5) Please send a completed 'Competing Interests' statement, including any COIs declared by your co-authors. If you have no competing interests to declare, please state "The authors have declared that no competing interests exist". Otherwise please declare all competing interests beginning with the statement "I have read the journal's policy and the authors of this manuscript have the following competing interests:"

**Reviewers' comments:**

Reviewer's Responses to Questions

**Comments to the Authors:**

**Please note here if the review is uploaded as an attachment.**

Reviewer #1: Keele et al. have used MS2 to generate large metabolomic, lipidomic, and oxylipin datasets in serum samples from a large and diverse cohort of 541 DO mice, and they have performed some genetic mapping on the resulting datasets. They have just under 1000 identified molecules, and a large set of 4500 unannotated/unidentified features. Many of these unannotated/unidentified features have significant differences across the cohort, so are presumably largely real metabolites/lipids, rather than noise or extremely-ephemeral intermediates. In general heritability of measurements is quite low (around 20%) for mouse cohorts, although certainly sufficient for this study given the sample size and high level of diversity. Interestingly (surprisingly, to me) many of the unannotated features _appear_ to be small peptides, or modified amino acids. There are also a handful of QTL hotspots, which is rather surprising to me that so many metabolites/lipids map to the exact same locus, and at such striking LOD scores.

Comments About Analysis & Interpretation

(1) You mention that aligning these results with other omics data in Do mice would be extremely useful to identify candidates of interest. For the mice in this study, are there any previous studies on the these exact same mice? Or are e.g. the liver datasets you mentioned both from completely separate DO cohorts? It looks like they are all from separate cohorts, meaning the animals you took plasma from here have never been analyzed before? I see now ref 22, that’s the same mice, but only trait data (it seems). Granted of course, for plasma analysis, it is difficult to overlay with tissue data, since – as mentioned – often no who knows what tissue the metabolite came from, or even if they do, what percentage of it came from which tissues, and how persistent it is.

(2) In Methods – and I also looked at your 2024 Blood paper – I don’t see it mentioned how the animals were controlled for their fast/fed state. Bile acids like TUDCA are going to radically change depending on the time since last meal, to the extent that it can be quite a challenge to get all mice on sufficiently-close feeding times such that its measurements can be particularly reliable, e.g. by timed fasting-refeeding. I don’t see it mentioned it in either paper, looking for keywords like “fast[ing]” and “fed” and from flipping through the methods.

(3) I certainly trust your QTL analyses, but I am still surprised at e.g. Fig 3A – that’s a huge number of lipids that map to chr8, like nearly all of them, and with massive LOD scores. Sure, the CES1 locus is important, but at those LOD scores and with that many lipids, it makes it seem like it is the master regulator of a huge range of lipids.

(4) Similar question for Fig 5A – that’s really a lot of metabolites mapping to the same locus with hugely-significant LOD scores, and Figure 6B, and Figure 7A. For 6B, the rationale that maybe it is MHC-related peptide fragments sounds reasonable to me, but the other hotspots are exceptionally ‘hot’. I don’t remember really ever seeing mQTL hotspots that hit such a large range of lipids with such large effect sizes. Normally what I expect is if you have, say, a knockout of a BCAA metabolizing enzyme, you see very striking buildups of a few particular metabolites, but then it eventually gets averaged out as you get further away from the key enzyme. What is the percent variance explained for e.g. LPC(22:6) in Figure 3C by the Cast allele? The effect is so large that I would expect e.g. a congenic line with just that region of Chr8 crossed into any of the other backgrounds to have just a massive impact on the levels of nearly all of those metabolites, even regardless of interacting genes – given anyway this is in DO mice. Are there any mice that are homozygous for Cast (or PWK or any of the other highly-striking alleles), and if so, do they have even more amplified effects? Or are there no such homozygotes, as maybe it would be embryonic lethal if so? (Can DOs be homozygotic at certain alleles? I don’t recall the breeding schema as to whether or not this is possible.)

(5) You show the heritability, but it would be nice to know how much variation is due to residuals, how much due to sex, etc. I guess this is already in your data (and maybe is in a supplemental table somewhere).

(6) Is there a data table that just has the abundances of all of the features in each of the mice? I see on your lab website things like the “dataset.lipids.stored.rds” but I’m not 100% sure this is the file I’m looking for. “dataset.metabolites.serum.rds” is that the other one I want? I downloaded it – it looks like probably yes. If that’s right, then that looks sufficiently easy to follow along.

(7) It would be nice to see a few more positive controls of expected outcomes – like do you expect kynurenine to go up in females? Is there a good metabolite in here that you could show as a convincing proxy of fasted/fed state? On MOFA, can you start to guess what factor 1-2-3-4 are? I assume one is genetics and one is residuals, probably another one is sex, but perhaps another one is fasted state? Is there any good way to figure that out? I see you have correlated them with the QTL hotspots, but within the list of features you measure, some may be characterized well enough to know “ah ha, this is a sex-linked metabolite”.

Comments About Typos / Formatting

(1) On page 10, you have “though these approaches are still subject to assumptions and multiple sources of uncertainty (e.g.,) .” The parenthetical “e.g.” is missing the example.

Summary

The authors have generated a large dataset in a diverse set of mice. I would like to know how the fasting/fed state was made consistent (again, perhaps I overlooked it in methods somewhere), particularly as I am surprised by how many features are mapping to the hot spots – the hot spots are blazing infernos both by their LOD score (especially for such a large cohort) and the number of features mapping to the same spot. This is a good (and massive) dataset and I agree with the analyses done. That said, given the surprise – to me – that so many metabolites map so significantly despite the (expected) moderate heritability, I think this paper would do better with a few more basic QC analyses at the start of the manuscript. For instance, surely by this point there are some metabolites that are already known to be elevated in PWK or CAST (or whatever) strain, and B6 certainly has many plasma features where it tends to be particularly low or particularly high. Similar as mentioned in comment #7, there must be some clear sex-linked metabolites (I guess you are showing these in Figure 1B). I see trimethylamineoxide is generally elevated in the plasma from women than from men. Seeing a list of examples like that, and those which are known to vary from strain (when such information is available) would help with interpreting the data.

But I think my only significant scientific concern is about the fasting of the mice, especially considering that males and females are batch-confounded, can you really be sure that e.g. TDCA is upregulated in females so dramatically, or whether it is that the cage changes or feeding or time or day or whatever was systematically slightly different in one cohort than the other? Fasting affects so many plasma metabolites so dramatically, but particularly the bile acids. Such huge QTL hotspots for such large ranges of metabolites make me wonder what alternate explanations could be, so I think showing additional basic examples of “this is an obesity-related metabolite, it is 2-fold up in mice that are > 40 g compared to those < 30g, voila.” would help quite a lot. Actually now that I think about it, I am kind of surprised that body weight is not used as a cofactor in any of these analyses – with such a large cohort of DOs there should be a pretty good range, and it should track closely with the levels of quite a few compounds.

In general I like this paper: I just would like clarification on the fast/fed state, and some additional ‘zoom in’ examples on specific metabolites that are known in literature, to serve as positive controls. I think that should be a pretty quick revision. Actually, I am now looking at the supplemental figures and Figure S4 is already along these lines. So… I guess I just want a bit more of that, and with your other key dependent and independent variables, somewhere around the end of Fig 1 or beginning of Fig 2.

Reviewer #2: The manuscript by Keele and colleagues provides a detailed analysis of the genetic regulation of metabolite levels in DO mice. Using a large population of DO mice and an comprehensive array of metabolomic platforms, the authors define a large landscape of metabolism under genetic regulation. A critical finding is the existence of mQTL hotspots in which multiple metabolites are associated with a single locus. The authors then characterize the hotspot loci using a series of analysis that utilize an extensive array of expression (eQTL) and protein (pQTL) data publicly available, and the authors provide a detailed assessment of candidate genes at the 7 hotspots identified. Overall, it is well written and data is presented clearly. There are some suggestions to improve some clarity for the reader.

• The authors have provided a significant amount of detail for the metabolomic assays and platforms. However, given the technical nature of metabolomics some additional information or edits would improve the clarity of the data generated. For example, the authors state in subsequent sentences: “Mass spectrometry for metabolites, Oxylipins, and lipids in red blood cells (RBCs) was described previously for this DO cohort [20,22,23]” and then “metabolomics extraction and analyses in 96 well-plate format were performed as described [56, 57]”. While the use of referenced methods is helpful, specific items such as mass spectrometry library size likely has increased over time and adding some additional information is helpful. For example, all 240 metabolites in table S1 have standards run as part of the process (from manuscript 58)? These are part of the single concentration standards used on each plate? A suggestion would be to expand or clarify some of the methods and if necessary, create a supplemental method section.

• Having some statements relaying the overall confidence of the matches to libraries would also be helpful. MS/MS and spectral matches do have some caveats and relaying these in the manuscript is appropriate as the nuances of this data type maybe foreign to readers of PLOS Genetics.

• What was the rationale to choose a 4Mb region to use for the hotspot analysis and why were 8 metabolites required to define a hotspot. Please include some rationale for this choice.

• The analysis of MS/MS spectra for protein and peptide moieties is novel. The result is a bit difficult to understand in the context of MHC in the results section. Adding some additional verbiage specific to Chr 17 hotspot would be helpful. Further in the same section, the ApoA2 locus is discussed. The result is intriguing given its somewhat complex effects on atherosclerosis and lipids and perhaps pleotropic effects on metabolism. The authors have used haplotypes at the ApoA2 locus to contrast specific metabolites under genetic regulation. Describing briefly the biological implications of the ApoA2 results in the discussion may better demonstrate the value of the data and approach taken.

• The title mentions carboxyl esterases as master regulators of circulating fatty acid metabolism, but this is only partially discussed in the manuscript. The title should be revised to better reflect the broad nature of the loci discussed. The manuscript contains several vignettes of data, and each are interesting but not solely related to carboxyl esterases nor fatty acids.

• For their figure 3, they're characterizing QTL allelic founder effects (tri-allelic, bi-alleleic) based off visual inspection of R/qtl2 BLUP plots. Are the authors are relying on collapsed haplotype effects (figure 3D, G, J) and identifying patterns of non-overlapping haplotype effect signal. The authors have created an alternative method to characterize alleles at a locus (TIMBR). Would use of this analytical method improve linkages between alleles and metabolites (similar to ApoA2 above)? Also, the last sentence of 2nd paragraph on page 4 is missing information- though these approaches are still subject to assumptions and multiple sources of uncertainty (e.g., ). What is missing after e.g.?

• In the methods (Sex differences) the authors mention " As mentioned above, sex is partially confounded with batch because animals were shipped in six separate sex-specific sub-cohorts.” The analysis of sexual dimorphism indicates 50% of the metabolites (~119) and 52% of lipids (~2897) were sex biased. The authors then state 14 of 15 metabolites and 26 of 29 lipid species were elevated levels in males. This statement seems to contradict the above. Are the authors referring to column G of the S1 table (pathway)? If so, revising this sentence to reflect this annotation is needed.

• The authors note that although they adjust for batch and use a stringent significance threshold, it is possible that confounding could still induce false positive sex differences. The authors should clarify how many of the mQTL identified are from metabolites that differ between sexes. Is there any concern that mQTL identified are sex specific? Most importantly are the mQTL identified at the hotspots robust to the confounding of batch and sex. This seems to be a limited number of mQTL so could a sex stratified analysis confirm the QTLs exist in both sexes even at a relaxed threshold?

• Their serum-to-RBC QTL overlap analysis does indicate tissue or cell type specificity of metabolism (and mQTL). This is an important point but the lack of mitochondria in RBC could explain some of these differences. Arguably, it would be better to compare different tissues or cells with similar organelles than the complex mixture of serum and RBC’s?

**********

**Have all data underlying the figures and results presented in the manuscript been provided?**

Large-scale datasets should be made available via a public repository as described in the *PLOS Genetics*  data availability policy, and numerical data that underlies graphs or summary statistics should be provided in spreadsheet form as supporting information.

Reviewer #1: Yes

Reviewer #2: Yes

**********

PLOS authors have the option to publish the peer review history of their article (what does this mean?). If published, this will include your full peer review and any attached files.

If you choose “no”, your identity will remain anonymous but your review may still be made public.

**Do you want your identity to be public for this peer review?**  For information about this choice, including consent withdrawal, please see our Privacy Policy.

Reviewer #1: No

Reviewer #2: No

[NOTE: If reviewer comments were submitted as an attachment file, they will be attached to this email and accessible via the submission site. Please log into your account, locate the manuscript record, and check for the action link "View Attachments". If this link does not appear, there are no attachment files.]

**Figure resubmission:**

**Reproducibility:**

To enhance the reproducibility of your results, we recommend that authors deposit laboratory protocols in protocols.io, where a protocol can be assigned its own identifier (DOI) such that it can be cited independently in the future. Additionally, PLOS ONE offers an option to publish peer-reviewed clinical study protocols. Read more information on sharing protocols at https://plos.org/protocols?utm_medium=editorial-email&utm_source=authorletters&utm_campaign=protocols

# Author comment: Author response to Decision Letter 1

**DOI:** 10.1371/journal.pgen.1012223.r003

29 Sep 2026

Attachment Submitted filename: Detailed responses to the Reviewers - 09232026.docx

# Peer review: Decision Letter 1

**DOI:** 10.1371/journal.pgen.1012223.r004

**Authors:** Zoltán Kutalik, Ana Viñuela

21 Oct 2026

Dear Dr D'Alessandro,

We are pleased to inform you that your manuscript entitled "Genetic architecture of the murine serum metabolome reveals carboxyl esterases as master regulators of circulating fatty acid metabolism" has been editorially accepted for publication in PLOS Genetics. Congratulations!

Before your submission can be formally accepted and sent to production you will need to complete our formatting changes, which you will receive in a follow up email. Please be aware that it may take several days for you to receive this email; during this time no action is required by you. Please note: the accept date on your published article will reflect the date of this provisional acceptance, but your manuscript will not be scheduled for publication until the required changes have been made.

Once your paper is formally accepted, an uncorrected proof of your manuscript will be published online ahead of the final version, unless you’ve already opted out via the online submission form. If, for any reason, you do not want an earlier version of your manuscript published online or are unsure if you have already indicated as such, please let the journal staff know immediately at plosgenetics@plos.org.

In the meantime, please log into Editorial Manager at https://www.editorialmanager.com/pgenetics/, click the "Update My Information" link at the top of the page, and update your user information to ensure an efficient production and billing process. Note that PLOS requires an ORCID iD for all corresponding authors. Therefore, please ensure that you have an ORCID iD and that it is validated in Editorial Manager. To do this, go to ‘Update my Information’ (in the upper left-hand corner of the main menu), and click on the Fetch/Validate link next to the ORCID field.  This will take you to the ORCID site and allow you to create a new iD or authenticate a pre-existing iD in Editorial Manager.

If you have a press-related query, or would like to know about making your underlying data available (as you will be aware, this is required for publication), please see the end of this email. If your institution or institutions have a press office, please notify them about your upcoming article at this point, to enable them to help maximise its impact. Inform journal staff as soon as possible if you are preparing a press release for your article and need a publication date.

Thank you again for supporting open-access publishing; we are looking forward to publishing your work in PLOS Genetics!

Yours sincerely,

Ana Viñuela

Academic Editor

PLOS Genetics

Zoltán Kutalik

Section Editor

PLOS Genetics

Aimée Dudley

Editor-in-Chief

PLOS Genetics

Anne Goriely

Editor-in-Chief

PLOS Genetics

www.plosgenetics.org

BlueSky: @plos.bsky.social

----------------------------------------------------

Comments from the reviewers:

Congratulations on your accepted manuscript. Please consider the comment from Reviewer #1 about the figure for comment #4 and ensure all documents and tables were included in the submission. Figures will be handle by the production team as per their request.

All the best,

Ana.

Reviewer's Responses to Questions

**Comments to the Authors:**

**Please note here if the review is uploaded as an attachment.**

Reviewer #1: Revision looks good to me. I actually quite like the figure added in the rebuttal for R#1 Comment #4 and I find that much easier / more complete to visualize than the conceptually similar figure panels in the actual paper - especially being able to see het/homo is nice. I don't actually recall seeing that exact visualization type for this exact data type before - but it's extremely intuitive. If you could fit that in the paper somewhere, I think it'd be nice.

Second off: I do not see "Document S1" with the supplemental figures in the revision file that is linked. I can only get the 4 supplemental XLSX tables for Data S1-S4, but NOT Document S1. The supplemental figures were included in the original submission - but I am not getting them here now in the revision. Anyway, I will believe the authors have done what they said they will, in which case I am fine with Figure S4 staying in supplemental.... even though I cannot see it. Probably it is the same or very similar to the original Figure S4, based on context.

Also on that note, the figures got horribly compressed and turned into very badly artifacted jpegs... the text is barely legible on many figure panels. In any case, this was not a problem with the initial submission, so I'm not sure what happened. Anyway, I am sure the editor will make sure that the original vector images are submitted when the paper actually goes out for publication.

No further comments - I thought the first submission was already very strong, so this was a quick re-review.

Reviewer #2: The authors have adequately addressed my previous queries. Their thoroughness in their responses is noted and appreciated.

**********

**Have all data underlying the figures and results presented in the manuscript been provided?**

Large-scale datasets should be made available via a public repository as described in the *PLOS Genetics*  data availability policy, and numerical data that underlies graphs or summary statistics should be provided in spreadsheet form as supporting information.

Reviewer #1: Yes

Reviewer #2: Yes

**********

PLOS authors have the option to publish the peer review history of their article (what does this mean?). If published, this will include your full peer review and any attached files.

If you choose “no”, your identity will remain anonymous but your review may still be made public.

**Do you want your identity to be public for this peer review?**  For information about this choice, including consent withdrawal, please see our Privacy Policy.

Reviewer #1: No

Reviewer #2: No

----------------------------------------------------

**Data Deposition**

If you have submitted a Research Article or Front Matter that has associated data that are not suitable for deposition in a subject-specific public repository (such as GenBank or ArrayExpress), one way to make that data available is to deposit it in the Dryad Digital Repository. As you may recall, we ask all authors to agree to make data available; this is one way to achieve that. A full list of recommended repositories can be found on our website.

The following link will take you to the Dryad record for your article, so you won't have to re‐enter its bibliographic information, and can upload your files directly:

http://datadryad.org/submit?journalID=pgenetics&manu=PGENETICS-D-26-00636R1

More information about depositing data in Dryad is available at http://www.datadryad.org/depositing. If you experience any difficulties in submitting your data, please contact help@datadryad.org for support.

Additionally, please be aware that our data availability policy requires that all numerical data underlying display items are included with the submission, and you will need to provide this before we can formally accept your manuscript, if not already present.

----------------------------------------------------

**Press Queries**

If you or your institution will be preparing press materials for this manuscript, or if you need to know your paper's publication date for media purposes, please inform the journal staff as soon as possible so that your submission can be scheduled accordingly. Your manuscript will remain under a strict press embargo until the publication date and time. This means an early version of your manuscript will not be published ahead of your final version. PLOS Genetics may also choose to issue a press release for your article. If there's anything the journal should know or you'd like more information, please get in touch via plosgenetics@plos.org.

# Editor report: Acceptance letter

**DOI:** 10.1371/journal.pgen.1012223.r005

**Authors:** Zoltán Kutalik, Ana Viñuela

PGENETICS-D-26-00636R1

Genetic architecture of the murine serum metabolome reveals carboxyl esterases as master regulators of circulating fatty acid metabolism

Dear Dr D'Alessandro,

We are pleased to inform you that your manuscript entitled "Genetic architecture of the murine serum metabolome reveals carboxyl esterases as master regulators of circulating fatty acid metabolism" has been formally accepted for publication in PLOS Genetics! Your manuscript is now with our production department and you will be notified of the publication date in due course.

The corresponding author will soon be receiving a typeset proof for review, to ensure errors have not been introduced during production. Please review the PDF proof of your manuscript carefully, as this is the last chance to correct any errors. Please note that major changes, or those which affect the scientific understanding of the work, will likely cause delays to the publication date of your manuscript.

Soon after your final files are uploaded, unless you have opted out or your manuscript is a front-matter piece, the early version of your manuscript will be published online. The date of the early version will be your article's publication date. The final article will be published to the same URL, and all versions of the paper will be accessible to readers.

For Research Articles, you will receive an invoice from PLOS for your publication fee after your manuscript has reached the completed accept phase. If you receive an email requesting payment before acceptance or for any other service, this may be a phishing scheme. Learn how to identify phishing emails and protect your accounts at https://explore.plos.org/phishing.

Thank you again for supporting PLOS Genetics and open-access publishing. We are looking forward to publishing your work!

With kind regards,

Anitha Samidurai

PLOS Genetics

On behalf of:

The PLOS Genetics Team

Carlyle House, Carlyle Road, Cambridge CB4 3DN | United Kingdom

plosgenetics@plos.org | +44 (0) 1223-442823

plosgenetics.org | Twitter: @PLOSGenetics
